# Effectiveness of a stepped-care programme of WHO psychological interventions in migrant populations resettled in Italy: Study protocol for the RESPOND randomized controlled trial

**DOI:** 10.3389/fpubh.2023.1100546

**Published:** 2023-01-25

**Authors:** Marianna Purgato, Giulia Turrini, Federico Tedeschi, Riccardo Serra, Lorenzo Tarsitani, Beatrice Compri, Giulia Muriago, Camilla Cadorin, Giovanni Ostuzzi, Pablo Nicaise, Vincent Lorant, Marit Sijbrandij, Anke B. Witteveen, José Luis Ayuso-Mateos, Roberto Mediavilla, Josep Maria Haro, Mireia Felez-Nobrega, Natasha Figueiredo, Giulia Pollice, David McDaid, A-La Park, Raffael Kalisch, Papoula Petri-Romão, James Underhill, Richard A. Bryant, Michela Nosè, Corrado Barbui

**Affiliations:** ^1^Department of Neuroscience, Biomedicine, and Movement Sciences, Section of Psychiatry, WHO Collaborating Centre for Research and Training in Mental Health and Service Evaluation, University of Verona, Verona, Italy; ^2^Department of Human Neurosciences, Sapienza University of Rome, Rome, Italy; ^3^Institute of Health and Society (IRSS) - UCLouvain, Brussels, Belgium; ^4^Department of Clinical, Neuro- and Developmental Psychology, WHO Collaborating Center for Research and Dissemination of Psychological Interventions, VU University, Amsterdam, Netherlands; ^5^Department of Psychiatry, Universidad Autónoma de Madrid (UAM), Madrid, Spain; ^6^Centro de Investigación Biomédica en Red de Salud Mental (CIBERSAM), Instituto de Salud Carlos III, Madrid, Spain; ^7^Department of Psychiatry, La Princesa University Hospital, Instituto de Investigación Sanitaria La Princesa (IIS-Princesa), Madrid, Spain; ^8^Instituto de Investigación Sanitaria del Hospital Universitario La Paz (IdiPAZ), Madrid, Spain; ^9^Research and Development Unit, Parc Sanitari Sant Joan de Déu, Barcelona, Spain; ^10^Sorbonne Université, INSERM, Institut Pierre Louis d'Epidémiologie et de Santé Publique, ERES, Paris, France; ^11^Care Policy and Evaluation Centre, Department of Health Policy, London School of Economics and Political Science, London, United Kingdom; ^12^Neuroimaging Center (NIC), Focus Program Translational Neuroscience (FTN), Johannes Gutenberg University Medical Center, Mainz, Germany; ^13^Leibniz Institute for Resilience Research, Mainz, Germany; ^14^Independent Research Consultant, Brighton, United Kingdom; ^15^School of Psychology, University of New South Wales, Sydney, NSW, Australia

**Keywords:** migrants, asylum seeker, refugee, psychological distress, COVID-19, resilience

## Abstract

**Introduction:**

Migrant populations, including workers, undocumented migrants, asylum seekers, refugees, internationally displaced persons, and other populations on the move, are exposed to a variety of stressors and potentially traumatic events before, during, and after the migration process. In recent years, the COVID-19 pandemic has represented an additional stressor, especially for migrants on the move. As a consequence, migration may increase vulnerability of individuals toward a worsening of subjective wellbeing, quality of life, and mental health, which, in turn, may increase the risk of developing mental health conditions. Against this background, we designed a stepped-care programme consisting of two scalable psychological interventions developed by the World Health Organization and locally adapted for migrant populations. The effectiveness and cost-effectiveness of this stepped-care programme will be assessed in terms of mental health outcomes, resilience, wellbeing, and costs to healthcare systems.

**Methods and analysis:**

We present the study protocol for a pragmatic randomized study with a parallel-group design that will enroll participants with a migrant background and elevated level of psychological distress. Participants will be randomized to care as usual only or to care a usual plus a guided self-help stress management guide (Doing What Matters in Times of Stress, DWM) and a five-session cognitive behavioral intervention (Problem Management Plus, PM+). Participants will self-report all measures at baseline before random allocation, 2 weeks after DWM delivery, 1 week after PM+ delivery and 2 months after PM+ delivery. All participants will receive a single-session of a support intervention, namely Psychological First Aid. We will include 212 participants. An intention-to-treat analysis using linear mixed models will be conducted to explore the programme's effect on anxiety and depression symptoms, as measured by the Patient Health Questionnaire—Anxiety and Depression Scale summary score 2 months after PM+ delivery. Secondary outcomes include post-traumatic stress disorder symptoms, resilience, quality of life, resource utilization, cost, and cost-effectiveness.

**Discussion:**

This study is the first randomized controlled trial that combines two World Health Organization psychological interventions tailored for migrant populations with an elevated level of psychological distress. The present study will make available DWM/PM+ packages adapted for remote delivery following a task-shifting approach, and will generate evidence to inform policy responses based on a more efficient use of resources for improving resilience, wellbeing and mental health.

**Clinical trial registration:**

ClinicalTrials.gov, identifier: NCT04993534.

## Introduction

The concept of migration refers to the process of moving from one country, region, or place to another one (1). As part of this concept, migration may occur within a country or across an international border, temporarily or permanently, and for a variety of reasons (2). The European Psychiatric Association guidance on mental health care of migrants has grouped reasons for migration in pull and push factors (1). Pull factors include educational or economic growth or personal factors, while push factors include political, poverty, terrorism, displacement, war or religious factors. Migrant is therefore an umbrella term, without any formal recognition under international laws, that generally includes a variety of different populations such as migrant workers, undocumented migrants, asylum seekers, refugees, internally displaced persons, and other populations on the move (1, 3).

Italy represents one of the first countries reached by migrants in the attempt to arrive in Europe. Over the last decades migration figures have fluctuated, with a peak in arrivals in 2016 (4). The number of arrivals reached 34 thousand in 2020 and almost 60 thousand in 2021 (4). UNHCR data indicates that, at the end of 2021, Italy hosted over 165 thousand forced migrants with a refugee status or requesting asylum (5). Most migrants arrived from Nigeria, Pakistan, Afghanistan, Mali, Somalia, and Gambia (4). According to the Italian reception system, after arrival, migrants are included in reception programmes that include food, housing, legal, and social guidance and support, and the development of individual interventions to promote socioeconomic inclusion and integration (6, 7).

During the migration process there may be factors that increase the vulnerability of individuals toward a worsening of subjective wellbeing, quality of life, and mental health, which, in turn, may increase the risk of developing mental health conditions. A discrepancy between expectations and achievements, poor support networks, difficulties in the processes of adjustment and acculturation, financial, administrative and legal issues, are commonly experienced stressors and living difficulties occurring during and after the migration process. Forcibly displaced migrants may additionally experience the loss of homes, hopes, possessions, and may be exposed to potentially traumatic events such as bombings, threats, captivity, torture, injury, and witnessing the death or injury of loved ones (8–11). After arrival in host countries major threats may include discrimination, economic problems, language barriers, loss of family and community support, poor access to social, educational and health services, and uncertain asylum application procedures (12). Notably, the COVID-19 pandemic has represented an additional stressor for migrant populations (13).

Epidemiological studies have documented that pre, during, and post migration stressors and potentially traumatic events are responsible for a high prevalence of psychological distress and mental health conditions in migrant populations, with differences related to reasons for migration, number and type of traumatic events, and time since resettlement (14–18).

In recent years, a growing number of randomized studies, and subsequently systematic reviews and meta-analyses, have documented the efficacy of psychological and psychosocial interventions on mental health outcomes in migrant populations, especially refugees and asylum seekers (19–21). However, these interventions require extensive training and considerable time to be delivered, staff with a mental health background, a monitoring and supervision infrastructure, and a face-to-face individual delivery modality in most cases. As these characteristics make them unlikely to be highly implemented, and unsuitable to address the needs of many people in a way that maximizes the use of resources, the World Health Organization (WHO) has developed a number of scalable psychological interventions for populations affected by adversity (22). A core feature of these interventions is that they can be trained and delivered by non-professional helpers, such as a trained peer, or helper at the workplace, or a psychosocial worker, following a task-shifting approach where tasks are moved from highly qualified health workers to health workers who have fewer qualifications in order to make more efficient use of the available resources (23). They are generally short in duration and highly protocolized, which makes them easy to be delivered by non-professional helpers. They have also been designed to be widely applicable to a variety of mental health problems irrespective of the presence of a mental health diagnosis, and easily adaptable to different populations, cultures and languages. Finally, the interventions and their implementation materials are open access, and they can be delivered through a variety of delivery modalities, including the use of digital technologies such as mobile phone or laptop or other.

In the present study, we designed a stepped-care programme of two scalable psychological interventions developed by the WHO and locally adapted for migrant populations. The first step consists of a mobile-supported website with a guided self-help programme, adapted from Self Help Plus (SH+), called Doing What matters in times of Stress (DWM) (24, 25). The second step is Problem Management Plus (PM+), an individual intervention based on problem-solving and cognitive behavioral therapy techniques delivered individually through video calls and offered only to participants who continue to show elevated levels of psychological distress after step 1 (26, 27). Both interventions have proved effective in humanitarian settings (28–35), but they have not been integrated into an online stepped-care programme.

## Study aim and hypothesis

The study aim is to evaluate the effectiveness and cost-effectiveness of the culturally and contextually adapted DWM/PM+ stepped-care programme among migrants resettled in Italy during the COVID-19 pandemic in terms of mental health outcomes (depression, anxiety and PTSD), resilience, wellbeing, self-identified problems, quality of life, and socio-economic impacts. We hypothesize a stronger decrease in anxiety and depression symptoms in the experimental arm, receiving the adapted DWM/PM+ stepped-care programme, as compared with the control arm, receiving care-as-usual (CAU).

## Methods and analysis

### Study design

The study is part of an EU-funded project named “Improving the Preparedness of Health Systems to Reduce Mental health and Psychosocial Concerns resulting from the COVID-19 Pandemic” (RESPOND) (www.respond-project.eu). Participants will be randomized to the adapted stepped-care DWM/PM+ intervention together with psychological first aid (PFA) or to PFA and CAU alone. Participants will self-report all measures at baseline before random allocation, 2 weeks after DWM delivery, 1 week after PM+ delivery and 2 months after PM+ delivery.

The present trial focuses on participants with a migrant background and elevated level of psychological distress, and it is coordinated by the WHO Collaborating Center of the University of Verona. We are currently recruiting participants through (a) key stakeholders such as non-governmental organizations (NGOs) located in Italy, (b) other community-based organizations offering legal and/or social and/or psychosocial support to this vulnerable group, or (c) targeted social media recruitment. Investigators proactively approached local organizations providing social, health, and/or legal support to migrant populations including refugees and asylum seekers to identify potentially eligible participants.

An Ethics and Data Advisory Board (EDAB) will monitor and provide expert advice on data management and all ethical, legal and societal issues that arise within the project, promoting integrity and a better alignment of RESPOND with social needs and expectations that may arise within or as a result of RESPOND. The study will be reported in accordance with the Consolidated Standards of Reporting Trials statement (36).

### Inclusion and exclusion criteria

Participants will be eligible to participate in the study if they meet the following criteria:

- 18 years or older;
- Being a migrant resettled in Italy temporarily or permanently (including migrant workers, undocumented migrants, asylum seekers, refugees, internationally displaced persons, or other persons on the move);
- Having elevated levels of psychological distress [Kessler Psychological Distress Scale (K10) >15.9] (37);
- Sufficient mastery (written and spoken) of one of the languages the DWM/PM+ intervention is being delivered in (English, Italian, French);
- Oral and written informed consent before entering the study.

Potential participants who meet the inclusion criteria will be excluded from participation in this study if they meet any of the following criteria:

- Acute medical conditions that require hospitalization;
- Imminent suicide risk, or expressed acute needs or protection risks that require immediate follow-up;
- Severe mental disorder (e.g., psychotic disorder);
- Severe cognitive impairment (e.g., severe intellectual disability or dementia);
- Initiated, stopped, or significantly modified psychiatric drug treatment over the previous 2 months;
- Currently receiving specialized psychological treatment (e.g., Cognitive Behavioral treatment, Eye Movement Desensitization and Reprocessing);
- Planning to permanently move back to their home country before the last quantitative follow-up assessment (2 months after PM+).

### Informed consent procedure and random allocation

Before being enrolled in the study, people who are interested will be informed using easily accessible language and terminology about the nature and scope of the study in a form understandable to them. A research assistant will explain the research and will provide the study materials. People agreeing to participate will be asked to complete a written consent form, offering a minimum consideration time of 1 week, before screening for inclusion and exclusion criteria. Participants meeting the inclusion criteria will be included in the randomized study. We will inform participants of the reasons why they can or cannot be included in the study. Participants will be asked to sign a second informed consent form, covering the optional recording of PM+ sessions, in case of PM+ administration. However, giving consent for the audio recordings will not be a condition for participating in the study. Audio recordings will only be used for fidelity assessments and supervisions.

Eligible participants will be randomly assigned with an equal probability of assignment to one of the two groups (allocation ratio 1:1). The randomization schedule will be generated using the web-based software Castor Electronic Data Capture (38). This electronic tool employs a variable block randomization method, in order to allocate groups randomly permuted in blocks of unequal size. The site investigators will not know the block size and will not be able to access the randomization list. In addition, the web-based software will allow random allocation only after the main information on the enrolled participant is entered, on verification of the inclusion criteria.

### Screening instruments

Psychological distress will be measures using the K10 (37). The K10 is a ten-item self-report questionnaire to screen broadly for psychological distress (e.g., anxiety and depression related distress) experienced in the past 30 days. Items are rated on a five-point Likert scale ranging from none of the time to all of the time. The sum of the ten items gives a total score ranging from 10 to 50. Higher scores represent higher levels of distress. The K10 has strong psychometric properties and has strong discriminatory power to distinguish DSM-IV cases from non-cases (37).

Suicidality will be explored with the ‘assessment of suicidal thoughts' risk tool from PM+ (26). Similarly, suspicion of a severe mental disorder and cognitive impairment will be assessed using the PM+ tool “Impairments possibly due to severe mental, neurological or substance use disorders” (26). This is a tool which is to be filled in by the assessor based on their observations and judgment of the client's behaviors. A judgment on four yes/no items that investigate the possibility of symptoms of severe mental, neurological, or substance use disorder is required. No questions are asked to the participant. The tool does not allow to make any diagnosis, but only indicates suspicion of a disorder.

### Interventions

Participants in the intervention arm will be offered a stepped care programme consisting of two scalable psychological interventions: DWM and PM+. All participants, both in the intervention and control group, will be offered a short counseling session, namely PFA, and will also maintain their care as usual.

The delivery of DWM, PM+ and PFA will be facilitated by peer helpers with at least primary school level literacy as well as good knowledge and skills in providing psychosocial support. Helpers will speak the same language as the participants (as well as being able to communicate in English). It is not necessary for helpers to have a psychosocial or mental health background in order to be able to offer these interventions. Necessary skills include foundational helping skills, such as effective community and rapport-building skills, and experience supporting people in distress. Helpers will receive training in delivering PFA, DWM, and PM+ by registered clinical psychologists trained by the WHO, and will receive continuous supervision throughout the study period.

### Psychological first aid

Before randomization, all participants will be offered individual Psychological First Aid (PFA) through a phone call or teleconferencing meeting with a research assistant (39). PFA is a WHO developed support strategy that involves humane, supportive and practical help for individuals who have been affected by serious humanitarian crises. PFA does not necessarily involve a discussion of the event(s) that cause the distress but aims particularly at five basic elements that are crucial to promote in the aftermath of crises, i.e., a sense of safety, calm, self- and community efficacy, connectedness, and hope. PFA consists of a conversation (up to 30–45 min) on various themes; in PFA, the helper provides non-intruding practical care and support, assesses needs and concerns, helps people to address basic needs (e.g., information), listens to people without pressuring them to talk, comforts and helps them to feel calm, as well as helping them to connect to information, services, and social support, and protects them from further harm.

### Care-as-usual (CAU)

In addition to PFA, both the intervention and control group will receive care-as-usual (CAU). CAU may include community care, social/legal support, psychoeducation, information about locally available referral options and about specific resources that might be helpful (e.g., hotlines for people in distress or experiencing loneliness, or support for women who might be experiencing gender-based violence).

### DWM/PM+ stepped care programme

Participants randomized to the intervention group will be offered DWM (24, 25). DWM is a booklet, adapted into a mobile website, divided into five monographic chapters covering five acceptance- and mindfulness-based strategies for managing stress. Chapters include audio recordings with different practices and exercises that help participants identify barriers and facilitators for practicing or triggers that exacerbate stress responses. During the local adaptation process undertaken in RESPOND, we adapted exercises to reflect barriers or stress triggers that might affect migrants resettled in Italy.

After allocation, DWM users will be assigned to a helper who will offer ongoing support with practical exercises and key concepts over the phone. An initial call will be arranged 2–5 days after entering the study. After that call, the participant receives a message with login details. The course is spread over 5 weeks, and new modules are released every week. Helpers also schedule weekly ongoing support calls. Support calls will take no longer than 15 min per call. The aim of the calls is to provide motivation and support in using the intervention. Participants who do not want to receive phone calls might contact their helpers using the messaging system included in the website. We will keep track of every helper-participant contact and use website metadata on participants' activity.

PM+ will be offered to participants reporting significant levels of psychological distress after DWM, as measured by a K10 score higher than 15.9. PM+ is a brief five-session psychological intervention based on cognitive behavioral therapy techniques (26, 27). Helpers will schedule 5 weekly sessions covering each strategy. As a result of the local adaptation process, we have adapted PM+ to be delivered online using teleconferencing tools, and shortened sessions from 90 to 60 min. Helpers will record the calls to monitor fidelity and go through identified barriers during practice over the week.

### Primary and secondary outcomes

Participants will be asked to complete online questionnaires at the following assessments: T0: screening for eligibility including psychological distress (before random allocation); T1: baseline assessment (before random allocation and PFA provision); T2: 2 weeks after DWM delivery; T3: 1 week after PM+ delivery; T4: 2 months after PM+ delivery. The primary outcome will be the change in symptoms of depression and anxiety from baseline to 2 months after the PM+ intervention, measured through the combined sum score of the Patient Health Questionnaire-9 (PHQ-9) (40, 41) and Generalized Anxiety Disorder (GAD-7) (42), previously validated as the PHQ-ADS (43).

In addition, the following measures will be considered secondary outcomes: depression (PHQ-9); anxiety (GAD-7); posttraumatic stress disorder (PCL-5) (44); self-identified problems (PSYCHLOPS) (45); resilience, operationalised as mental health after stressor exposure (46); quality of life (EQ-5D-5L) (47); resource use and economic outcomes, collected using a modified version of the Client Service Receipt Inventory (CSRI) (48). The CSRI includes information on the use of healthcare and other services, time out of employment and other usual activities and the need for informal care.

Additional study parameters include the following: demographic data; COVID-19 exposure questionnaire; Positive Appraisal Style Scale, content focused (PASS-content) (46); exposure to adverse life-events (BTQ questionnaire) (49); treatment fidelity (DWM: tracking app usage based on meta-data, PM+: audio records, checklists); satisfaction (qualitative assessment); acceptability of the programme (qualitative assessment); adverse events; implementation indicators: reach, dose, resource use, costs of recruiting, training and retaining staff delivering the stepped-care programme, programme costs, adaptation, and quality. An overview of measures and the time periods when they are collected is presented in Table 1.

**Table 1:** Overview of concepts, measures, type of study parameter and time periods when they are collected.

**Concept**	**Measures**	**Type of study parameter**	**Time period**						
			**Screening (T0)**	**Baseline (T1)**	**DWM**	**Post-assessment (T2)**	**PM+**	**Post-assessment (T3)**	**Follow-up assessment (T4)**
Psychological distress	K10	Screener	x			x			
Suicide risk:									
Face-to-face *or*	PM+ tool	Screener	x			x	x		
Self-administered	Step-by-step question	Screener	x			x		x	x
Mental, neurological or substance use disorders	PM+ tool	Screener	x						
Depression and anxiety:	PHQ-ADS	Primary							
Subscale depression	PHQ-9	Secondary		x		x		x	x
Subscale anxiety	GAD-7	Secondary		x		x		x	x
Posttraumatic stress reactions	PCL-5	Secondary		x		x		x	x
Self-identified problems	PSYCHLOPS	Secondary		x		x		x	x
Resilience	MIMIS	Other		x		x		x	x
Quality of life	EQ-5D-5L	Secondary		x		x		x	x
Impact on resource use/costs	CSRI	Secondary		x		x		x	x
Socio-demographics		Other		x					
COVID-19 exposure	Questionnaire	Other		x		x		x	x
Resilience factors	PASSc	Other		x		x		x	x
Exposure to life events	BTQ	Other		x				x	
Treatment fidelity:									
DWM	Metadata	Other			x				
PM+	Audio records	Other					x		
Satisfaction DWM	CSQ Interview	Other				x			
Satisfaction PM+	CSQ Interview	Other						x	

### Measures

#### Patient health questionnaire: Anxiety and depression scale (PHQ-ADS)

The PHQ-ADS is a 16-item self-reported instrument that combines the nine-item Patient Health Questionnaire depression scale (PHQ-9) and seven-item Generalized Anxiety Disorder scale (GAD-7) into a composite measure of depression and anxiety (43). Respondents are asked about how much each symptom has bothered them over the past 2 weeks, with response options of “not at all,” “several days,” “more than half the days,” and “nearly every day,” scored as 0, 1, 2, and 3. The scale can range from 0 to 48, with higher scores indicating higher levels of depression and anxiety symptoms.

#### PTSD checklist for DSM-5 (PCL-5): Eight-item version

The eight-item PCL-5 is a self-reported instrument that measures PTSD symptoms (44). Respondents are asked how much each symptom has bothered them over the past 4 weeks, with response options of “not at all,” “a little bit,” “moderately,” “quite a bit,” and “extremely.” Items are rated on a 0–4 scale. The scale can range from 0 to 32 for the 8-item version, with higher scores indicating higher levels of PTSD symptoms.

#### PSYCHLOPS: Self-identified problems

The Psychological Outcomes Profiles (PSYCHLOPS) scale is a patient-generated outcome measure as an indicator of change after therapy (45). PSYCHLOPS consists of three domains: problems (two questions), function (one question), and wellbeing (one question). Participants are asked to give free text responses to the problem and function domains. Responses are scored on an ordinal six-point scale producing a maximum score of 18 (six points per domain).

#### Exposure to stressors

Resilience can be defined as maintaining or recovering good mental health after facing adversity (46), which requires collecting information on mental health symptoms and exposure to stressors. To this end, a new measure of stressor exposure was developed, aimed to assess stressors particular to the study population. This measure was based on an adaptation of the Mainz Inventory of Micro stressors (MIMIS) (50), an objective measure of micro-stressors or daily hassles. After the COVID-19 outbreak, a shorter version, including pandemic-related stressors, was developed (51). In RESPOND qualitative interviews of the target population further informed which stressors participants were likely to encounter. The resulting measure consists of 22-item adaptation that includes: three general life events (e.g., recent break-up); six everyday stressors (e.g., excessive workload, financial problems); five COVID-19-specific stressors (e.g., being forced to quarantine); and eight refugee and migrant population specific stressors [e.g., Lack of access to services (for example: health services, etc.)]. Participants are asked to rate general life events that occurred in the past 2 months on a 5-point Likert scale, ranging from 0 (“never happened”) to 4 (“it had a major impact on me”). The remaining 19 items are rated on a 4-point Likert scale, ranging from 0 (“did not happen/almost never”) to 3 (“every day or nearly every day”). These items ask about the last 14 days.

#### EuroQol 5-dimensional descriptive system: 5-level version (EQ-5D-5L)

The EQ-5D-5L consists of the EQ-5D and the EQ-Visual Analog Scale (VAS) (47). It rates the level of impairment across five dimensions: mobility, self-care, usual activities, pain/discomfort, and anxiety/depression. Each dimension has five levels: none, slight, moderate, severe, and extreme problems. The labels for the 5L followed the format “no problems,” “slight problems,” “moderate problems,” “severe problems,” and “unable to”/“extreme problems” for all dimensions. The EQ-VAS, is measured from 0 (the worst imaginable health, not necessarily death) to 100 (the best imaginable health state). The endpoints of the scale are called “The best health you can imagine” and “The worst health you can imagine,” and the current health status of that day needs to be indicated, after which the number checked on the scale also needs to be written down. Higher scores indicate better quality of life.

#### Client service receipt inventory (CSRI)

An adapted version of the CSRI in Italian will be used to estimate changes in resource use. Appropriate unit costs will be used to estimate the costs of this resource used estimate. The RESPOND-adapted version used in Italy consists of a 13-item self-reported instrument that asks about the number and duration of contacts with healthcare professionals (physicians, mental health specialists, and nurses) in the past 2 months. It collects data on service utilization (e.g., use of health care and other services, time out of employment and other usual activities, need for informal care) and related characteristics of people with mental health problems.

#### COVID-19 exposure questionnaire

The COVID-19 exposure questionnaire includes 11 questions related to the impact of COVID-19. The questionnaire is based on other COVID-19 questionnaires. It will be administered at each time-point.

#### Positive appraisal style scale – content focused (PASSc)

The PASS-content is a measure of a person's general tendency of how they appraise stressors that they encounter; their positive appraisal style (PAS) (46). The 12-item questionnaire, asks the participant to report how frequently they have specific thoughts when facing adversity on a 4-point Likert scale from 1 = never to 4 = (almost) always (e.g., “I think that the situation also has its positive sides”). This measure was included at each time-point.

#### Brief trauma questionnaire

The Brief Trauma Questionnaire (BTQ) is a brief self- report questionnaire that is derived from the Brief Trauma Interview (49). The BTQ was originally designed to assess traumatic exposure according to DSM-IV but specifically asked only about Criterion A.1 (life threat/serious injury) because of the difficulty of accurately assessing A.2 (subjective response) in a brief self-report format. Criterion A.2 has been eliminated from the PTSD diagnostic criteria in DSM-5, so the BTQ provides a complete assessment of Criterion A. The questionnaire may be used to determine whether an individual has had an event that meets the A Criterion, or to determine the different types of Criterion A events an individual has experienced.

#### Adverse events and serious adverse events

Adverse events are defined as any undesirable experience occurring to a participant during the study, whether or not considered related to the trial procedure or the stepped-care DWM/PM+ intervention. All adverse events reported spontaneously by study participants or observed by the investigators will be recorded and reported to the EDAB.

#### Other measures

We will conduct in-depth interviews with key informants to assess the feasibility of programme implementation. We will select informants among completers and non-completers of DWM and PM+ interventions. We will also conduct interviews and focus group interviews with participants' relatives.

### Masking

Masking participants and helpers about the intervention status will be impossible, due to the nature of the intervention. In addition, as the study does not include any observer-reported outcomes, it cannot include any masked outcome assessor. However, the statistician performing the analyses will be masked to the participants' allocation.

### Sample size and power calculations

Based on prior studies on PM+ (32, 52), we aim to detect a small to medium Cohen's d effect size of 0.3 in the PM+ group at 2 months post-intervention based on the primary composite outcome PHQ-ADS. A power calculation for a repeated measurement design suggests a minimum sample size of *N* = 74 per group (power = 0.95, alpha = 0.05, two-sided, rho = 0.9) to identify an effect at the time of interest. Assuming an attrition rate of 30%, we aim to include a total number of 212 participants [106 in the stepped-care DWM/PM+ intervention group (with PFA and CAU) and 106 in the PFA and CAU comparison group].

### Statistical analysis

All primary and secondary analyses will be performed on an intention-to-treat (ITT) basis. The ITT population will consist of all participants randomly assigned to the competitive intervention strategies and with at least data on the baseline assessment available. In order to check the robustness of results, all outcomes will be additionally analyzed using a per protocol (PP) approach, that will include only DWM participants clicking through all of the content of at least 3 modules (regardless of whether or not they engage in phone calls/messages with the helper) and PM+ participants attending at least 4 sessions. The analysis of the PP population will be used for confirmatory purposes only. If < 5% of participants do not receive the allocated intervention according to the study protocol, the PP analysis will not be performed.

The primary outcome will be summarized using number of subjects (*n*), minimum and maximum; and means, standard deviations (SD) for normally distributed data, or medians and inter-quartile ranges for non-normally distributed data. To compare the two treatment groups at baseline, standardized mean differences will be calculated. The primary analysis will simultaneously assess treatment effect on the average PHQ-ADS score at each time-point in the ITT population. The main conclusion of the trial will be based on the ITT analysis of the primary outcome (i.e., the effect on PHQ-ADS score at the 2-month follow-up). To estimate the treatment effect for the time-points T2, T3, and T4, a linear mixed model will be employed for the analysis on PHQ-ADS, which will have time as a fixed effect, baseline measurement of PHQ-ADS as covariate, and subject as random effects. Our model will be re-parametrized by constraining the treatment fixed-effect to be 0, and by including a time^*^treatment interaction at T2 as well. In this way, in each time-point, the treatment effect will be measured as the interaction between time (as a categorical variable) and treatment, with its value at T4 being our outcome of interest. The mean difference between two treatment arms at each visit/time together with its 95% confidence interval will be derived from the mixed model. In addition, a covariate-adjusted mixed model of primary outcome will be performed by adding covariates showing imbalance at baseline (as measured by a Standardized Mean Difference above 0.1 in absolute value). Robust standard errors will be used in all models.

A secondary analysis of the effect of treatment on PHQ-ADS score will be conducted using the per protocol (PP) population, using the same approach as reported above. In addition, a covariate-adjusted mixed model of primary outcome will be performed using the PP population by adding pre-specified covariates at baseline (gender, age, education, prior trauma, COVID-19 related events and the stressor exposure).

Missing data will be treated as missing at random (MAR). No imputations of missing values will be made, as multilevel models can deal with missing data (53). In the case only some items are missing for a specific scale, we will perform the Corrected Item mean Substitution method (i.e., the item mean across participants weighted by the subject's mean of completed items) (54), using information from subjects belonging to the same treatment arm for the same follow-up time (estimated values above the maximum or below the minimum admissible value will be set to maximum/minimum).

A linear mixed model with robust standard errors, as mentioned for the primary analysis, will be carried out to analyse the following secondary outcomes: depressive symptoms (PHQ-9), generalized anxiety (GAD-7); posttraumatic stress reactions (PCL-5), self-identified problems (PSYCHLOPS), and quality of life (EQ-5D-5L), as well as outcome-based resilience, operationalised as the PHQ-ADS total score against stressor exposure count (55). The secondary outcomes will be analyzed on the ITT population only. Further, mediation analyses including positive appraisal style (PASS-content) and the primary and secondary outcomes will be conducted.

Health economic analysis will be conducted from both a health care and societal perspective. It will determine the cost effectiveness of intervention compared to care as usual over the study period. The primary outcome measure for the economic analysis will be incremental cost per QALY gained at 2-month follow-up. This involves synthesis of data on quality of life using an Italian specific valuation of health states, as well as data on the costs of interventions and subsequent resource utilization in the two trials. Between-group comparison of mean costs will be completed using appropriate statistical tests depending on the type and distribution of data. Univariate sensitivity analyses, using non-parametric bootstrapping to account for uncertainty around cost and effectiveness in trial parameters, will be performed by varying the costs of interventions. Cost effectiveness planes and cost effectiveness acceptability curves will be presented.

## Discussion

The results of this randomized trial will be considered together with the results of three other trials that are being conducted using the same study design, but focussing on different vulnerable groups, as part of the RESPOND programme. While the present study has a focus on migrants including refugees and asylum seekers resettled in Italy, health-care workers is the target population of a study conducted in Spain (56), people living in social adversity conditions are being recruited in France, and in the Netherlands labor migrants are the target population. Taken together, these studies will quantify the beneficial effects of stepped-care psychological interventions in a wide range of populations exposed to COVID-19 related psychological distress or other types of stressors, and will make available DWM/PM+ packages adapted for remote delivery following a task-shifting approach. Through wide dissemination of the results of these studies, we aim to suggest policy responses based on a more efficient use of resources for improving resilience, wellbeing and poor mental health. Our vision is that these stepped care psychological interventions could be integrated into holistic response to future COVID-19 waves or future epidemics or health crises related to war or other humanitarian or economic emergencies.

As part of the RESPOND programme, studies conducted across vulnerable groups will be combined to reliably detect predictors and moderators for the effects of the DWM/PM+ programme. This is expected to generate unique information for future personalized delivery of mental health care.

## Ethics and dissemination

The protocol, informed consent form, procedure to obtain consent, and the procedure to protect confidentiality of personal data of this trial was approved the Comitato Etico per la Sperimentazione Clinica delle province di Verona e Rovigo, Approval ID 46725 of 10/08/2021, and are registered in Clinicaltrial.gov (ClinicalTrials.gov Identifier: NCT04993534). Any amendments on the protocol will be communicated though updating in the public webpage of the Trial Registry. The funder has no role in study design; collection, management, analysis, and interpretation of data; writing of the report; and the decision to submit the report for publication. The results of this trial will be published in peer-reviewed journal articles and the final trial dataset will be made available after de-identification of the participants.

All data will be handled confidentially and will be coded by a code known only by the research team. Processing of personal data will comply to the General Data Regulation (“GDPR”) on the protection of individuals regarding the processing of personal data and the free movement of such data. Data including personal information will be stored in a locked record at the WHO Collaborating Center of the University of Verona to ensure the confidentiality of the study participants. Only authorized research personnel will have access to this data. According to the data management rules of RESPOND, all partners acknowledge and agree that no personal data, as defined in Regulation (EU) 2016/679 of the European Parliament and of the Council of 27 April 2016 on the protection of natural persons with regard to the processing of personal data and on the free movement of such data, and repealing Directive 95/46/EC (General Data Protection Regulation) (“GDPR”), will be exchanged between the Parties. Moreover, all partners in RESPOND acknowledge and agree that each partner is considered independent controller, as defined in GDPR, for its processing of personal data and will act in accordance with applicable data protection laws (including but not limited to GDPR).

## Ethics statement

The study protocol was approved by the Comitato Etico per la Sperimentazione Clinica delle province di Verona e Rovigo, Approval ID 46725 of 10/08/2021. The patients/participants provided their written informed consent to participate in this study.

## Author contributions

CB and MP drafted this manuscript, and all authors contributed to review and editing. All authors contributed to conceptualization, methodology, data curation, and protocol development. All authors approved the final version.
